# The Prevalence and Correlates of Arterial Stiffness in Patients With Treated Hypertension: Oscillometric Pulse Wave Analysis During 24‐h Ambulatory Blood Pressure Monitoring

**DOI:** 10.1155/ijhy/5824092

**Published:** 2026-02-10

**Authors:** Damla Tufekci, Tuncay Sahutoglu, Ekrem Kara

**Affiliations:** ^1^ Department of Endocrinology and Metabolism, Recep Tayyip Erdogan University Faculty of Medicine, Rize, Türkiye, erdogan.edu.tr; ^2^ Department of Nephrology, Mehmet Akif Inan Training and Research Hospital, Sanliurfa, Türkiye; ^3^ Department of Nephrology, Recep Tayyip Erdogan University Faculty of Medicine, Rize, Türkiye, erdogan.edu.tr

**Keywords:** ambulatory blood pressure monitoring, arterial stiffness, oscillometric pulse wave analysis, treated hypertension

## Abstract

**Background:**

We investigated the prevalence and correlates of arterial stiffness in treated hypertension using oscillometric pulse wave analysis during 24‐h ambulatory blood pressure monitoring (ABPM).

**Methods:**

In this single‐center cross‐sectional study, 131 patients (median age 51.0 years, range 17.0–86.0; 54.2% female) underwent 24‐h ABPM. Measurements included 24‐h, daytime, and night‐time SBP, DBP, MAP, pulse pressure, dipping status, and estimated pulse wave velocity (ePWV) derived by the Mobil‐O‐Graph (ARCSolver; age/SBP‐dependent).

**Results:**

High ePWV (> 9 m/s) was present in 16.8% of patients. Compared with low/moderate ePWV, the high‐ePWV subgroup was older (*p* < 0.001) and had higher FPG (*p* < 0.001), higher creatinine and lower eGFR (both *p* < 0.001), greater proteinuria (*p* = 0.006), and a lower frequency of systolic dipper status (*p* = 0.033). In simple correlations, 24‐h ePWV was correlated positively with 24‐h, daytime, and night‐time values of DBP, MAP, and pulse pressure, BMI, FPG, creatinine, uric acid, and proteinuria, and negatively with systolic dipping, diastolic dipping, albumin, and eGFR. However, after adjusting for age, age^2^, and 24‐h SBP, the partial correlation analysis revealed that ePWV was negatively correlated only with FPG (*r* = −0.216, *p* = 0.014) and hsCRP (*r* = −0.220, *p* = 0.031) and positively correlated only with total cholesterol (*r* = 0.243, *p* = 0.043) and LDL (*r* = 0.359, *p* = 0.004).

**Conclusion:**

Elevated ePWV identifies a high‐risk phenotype in treated hypertension, characterized by advanced age and renal impairment. However, these associations appear intrinsic to the algorithm’s reliance on age and SBP. After adjusting for these inputs, ePWV did not exhibit independent associations with clinical parameters, suggesting it should be viewed as an integrated derivative of age and blood pressure rather than a separate physiological measure. ePWV values should be interpreted with caution, recognizing their inherent dependence on algorithmic inputs.

## 1. Introduction

Arterial stiffness plays an important role in the pathogenesis of hypertension as well as in predicting the risk of subclinical vascular disorders, target organ damage, and future cardiovascular events in hypertensive patients [1, 2]. Age, hypertension, and arterial stiffness are involved in a “feed‐forward” relationship [1, 3, 4]. Arterial stiffness causes a progressive age‐related increase in systolic blood pressure (SBP), while hypertension exacerbates the process of vascular aging and serves as a precursor of arterial stiffness by causing diminished arterial elasticity and compliance [4–7].

The early detection of arterial stiffness is increasingly recognized as a valuable tool for initiating preventive measures and guiding optimal treatment against the progression of vascular damage and in predicting, staging, and managing cardiovascular risks in hypertensive patients [1, 2, 8–10].

Pulse wave velocity (PWV), the major component of pulse wave analysis (PWA), is an established gold standard for measuring arterial stiffness, which is intrinsically linked to the elasticity of the arterial vessels [6, 8, 10]. PWV is considered a key biomarker of clinical importance in cardiovascular diagnostics, yielding deep insights into vascular health and cardiovascular risk, particularly in monitoring the progression of hypertensive vascular disease [10–12].

Oscillometric brachial PWA has emerged as a noninvasive and reproducible oscillometric method that allows the measurement of peripheral and central blood pressure and PWA parameters such as PWV [10, 13–16]. Modern ambulatory blood pressure monitoring (ABPM) devices use specific algorithms and software for PWA and provide conventional 24‐h ABPM data in addition to the measurement of PWV through oscillometric brachial PWA [9, 17, 18]. The Mobil‐O‐Graph device is a user‐friendly and lower‐cost tool that combines cuff oscillometry and PWA to measure oscillometric ePWV during 24‐h ABPM [5, 15, 16, 19]. Mobil‐O‐Graph oscillometric ePWV results have been validated against intra‐aortic catheter measurements and exhibit strong correlation with the gold‐standard, carotid femoral PWV (cfPWV) [14, 15, 20].

This study investigated the prevalence and predictors of arterial stiffness in patients with treated hypertension, using oscillometric PWA provided by a Mobil‐o‐Graph device.

## 2. Materials and Methods

### 2.1. Study Population

One hundred and thirty‐one patients (median age 51.0 years, range 17.0–86.0 years, 54.2% female) with treated hypertension were included in this single‐center cross‐sectional study conducted at the nephrology and endocrinology clinics of a tertiary care hospital. Patients with essential hypertension who had been receiving stable antihypertensive treatment for at least 3 months were included. Exclusion criteria were the presence of secondary hypertension, major cardiovascular or renal disease, severe organ failure, urinary tract obstruction, rheumatological disease, malignancy, and pregnancy.

Written informed consent was obtained from each participant following a detailed explanation of the study objectives and protocol. The research was conducted in accordance with the ethical principles set out in the Declaration of Helsinki and was approved by the Recep Tayyip Erdogan University clinical research ethics committee (no. 148, dated 11/07/2018).

### 2.2. Statement on the Use of Generative Artificial Intelligence

The authors declare that they have not used any type of artificial intelligence generated content (AIGC) tools such as ChatGPT and others based on large language models (LLMs) in developing any portion of this manuscript.

### 2.3. Assessments

Data concerning demographics (age and gender), body mass index (BMI), smoking status (defined as current cigarette use), comorbid diabetes, antihypertensive medications, and baseline blood biochemistry findings were recorded for each patient. Past smokers and users of other tobacco forms were classified as nonsmokers, as noncigarette use was extremely rare in this population. The 24‐h ABPM measurements included 24‐h total, daytime, and night‐time data for SBP, diastolic BP (DBP), mean arterial pressure (MAP) and pulse pressure, nocturnal dipping, dipper status (systolic and diastolic), and ePWV (m/s; 24‐h total, daytime, and nighttime). Demographic, clinical, and laboratory findings, as well as 24‐h ABPM measurements (total, daytime, night‐time BP, dipping, and dipper status), were compared across three ePWV subgroups, low (< 6 m/s), moderate (6–9 m/s), and high (> 9 m/s). The high‐ePWV cut‐off was prespecified as > 9 m/s to reconcile guideline and outcome evidence: the 2018 ESC/ESH Guidelines recommend ≥ 10 m/s as a conservative marker of subclinical organ damage, whereas recent outcome‐based analyses support > 9 m/s as a clinically relevant threshold for increased arterial stiffness [21]. Accordingly, we adopted the > 9 m/s threshold as a pragmatic and evidence‐informed criterion to identify individuals with arterial stiffness within our treated hypertensive cohort [22, 23]. Correlations between 24‐h total ePWV values and the study parameters (age, BMI, blood biochemistry, and 24‐h ABPM values) were also analyzed.

### 2.4. Oscillometric PWA

A Mobil‐O‐Graph (PWA‐EMI GmbH, Stolberg, Germany) was used for oscillometric PWA and 24‐h ABPM. This commercially available brachial oscillometric ABPM device has been validated in accordance with the European Society of Hypertension (ESH) recommendations [5, 15, 16, 19]. The device records brachial pulse waves (instantaneously) and acquires measurements during the diastolic phase for ∼10 s using a conventional brachial cuff with a high‐fidelity pressure sensor; oscillometric brachial SBP/DBP is used to calibrate the brachial waveforms. PWV is derived by the ARCSolver algorithm (proprietary), which generates the aortic waveform [5, 19, 24]; the Mobil‐O‐Graph software (HMS) then performs wave‐separation analysis, decomposing the aortic waveform into forward and backward traveling waves based on a physiological aortic flow waveform [25]. Importantly, the PWV values represent estimated PWV (ePWV) rather than direct hemodynamic measurements; because ARCSolver incorporates age and brachial SBP into its calculation model, this dependency was considered when interpreting correlations between PWV and clinical variables.

For the 24‐h ABPM protocol, participants received appropriately sized cuffs and fresh batteries. Recordings were initiated at 09:00 and concluded at 09:00 the following day, with measurements every 15 min during the day (07:00–22:00) and every 30 min at night (22:00–07:00). Quality control consisted of visual assessment of the curves on the Mobil‐O‐Graph clinical report screen. Patient instructions followed the 2014 ESH Practice Guidelines [26]: patients kept a diary card; recorded major events during monitoring; documented the name, dose, and timing of each prescribed drug; noted sleeping and waking times and times of main meals; and briefly described any unusual activity or symptoms. They were instructed not to interfere with the device during monitoring. Nondipping was defined as < 10% nocturnal reduction in either SBP or DBP. ABPM data were printed and saved for parameter extraction. All patients included in the final analysis met ESH ABPM validity criteria: ≥ 70% successful readings, ≥ 20 daytime readings, and ≥ 7 night‐time readings [26]. Furthermore, the aggregated SBP values reported were derived exclusively from those oscillometric readings where ePWV was successfully computed by the ARCSolver algorithm.

### 2.5. Blood Biochemistry

Laboratory assessments included the measurement of various blood biochemistry parameters using the spectrophotometric method (Beckman Coulter AU680): fasting plasma glucose (FPG; 74–100 mg/dL), hemoglobin (males: 12.89–16.73, females: 11.33–14.56 g/dL), creatinine (males: 0.67–1.17, females: 0.51–0.95 mg/dL), uric acid (3.5–7.2 mg/dL), albumin (35–52 g/L), sodium (135–146 mmol/L), potassium (3.5–5.1 mmol/L), calcium (8.8–10.6 mg/dL), phosphorus (2.5–4.5 mg/dL), total cholesterol (120–200 mg/dL), low‐density lipoprotein (LDL; < 160 mg/dL), triglyceride (50–150 mg/dL), high‐sensitivity C‐reactive protein (hs‐CRP; 1–3 mg/dL), and proteinuria (0–300 mg/g). Additionally, intact parathormone (PTH; pg/mL) and ferritin (ng/mL) were measured using the immunoassay method (Siemens Advia XPT), while estimated glomerular filtration rate (eGFR; mL/min/1.73 m^2^) values were also recorded.

### 2.6. Statistical Analysis

Statistical analysis was performed using IBM SPSS Statistics for Windows, version 26.0 software (IBM Corp.2019, Armonk, NY, USA). The Kolmogorov–Smirnov/Shapiro–Wilk test was used to investigate normality of distribution. The chi‐squared (*χ* ^2^) and Fisher’s exact tests were used to compare categorical data, while the Kruskal–Wallis test was applied to compare three independent non‐normally distributed variables.

In comparing categorical data by group, the chi‐squared test was used if all expected observation values were greater than 5, and the Fisher exact test was used if any of the expected observation values ​​were less than 5. Multiple comparisons of proportions were examined using the *Z* test with the Bonferroni correction. To address the algorithmic dependence of the Mobil‐O‐Graph device, partial correlation coefficients were calculated to analyze the relationships between ePWV and quantitative demographic and clinical variables after controlling for age, age^2^ (to account for nonlinear effects), and SBP. Partial correlation analyses were performed separately for each variable including only the observations without missing data (listwise deletion). Data were expressed as mean ± standard deviation (SD), median, interquartile range (IQR, 25%–75%), minimum–maximum, and percentage (%) values, as appropriate. *p* < 0.05 was considered statistically significant.

## 3. Results

### 3.1. Baseline Characteristics

The patients’ median age was 51 years (range 17.0–86.0), and 54.2% were women. Active smoking and comorbid diabetes were present in 19.8% and 21.3% of the patients, respectively (Table 1). Calcium channel blockers were the most frequently prescribed antihypertensive regimen (47.7%) (Table 1). Blood biochemistry results are summarized in Table 1.

**TABLE 1:** Demographic, clinical, laboratory features: overall and across the PWV subgroups.

	Total (*n* = 131)	Pulse wave velocity groups			*p* value
		Low (*n* = 37)	Medium (*n* = 72)	High (*n* = 22)	
Demographics					
Age (years), median (min–max)	51.0 (17.0–86.0)	24 (17–43)^a^	52 (37–65)^b^	73 (60–86)^c^	**< 0.001**
Gender, *n* (%)					0.580
Male	71 (54.2)	15 (40.5)	33 (45.8)	12 (54.5)	
Female	60 (45.8)	22 (59.5)	39 (54.2)	10 (45.5)	
BMI (kg/m^2^), median (min–max)	30.5 (20.0–45.1)	27 (20–40.8)^a^	31 (22–45.1)^b^	30.3 (21–43)^ab^	**0.005**
Smoking, *n* (%)	26 (19.8)	8 (21.6)	16 (22.2)	(9.1)	0.394
Comorbid diabetes, *n* (%)	28 (21.3)	1 (2.7)^a^	20 (27.8)^b^	7 (31.8)^b^	**0.001**
Antihypertensive treatment, *n* (%)					
CCB	62 (47.3)	15 (40.5)	37 (51.4)	10 (45.5)	0.551
Beta‐blocker	36 (27.4)	8 (21.6)	24 (33.3)	4 (18.2)	0.243
ARB	32 (24.4)	10 (27)	14 (19.4)	8 (36.4)	0.252
ACEi	34 (25.9)	10 (27)	21 (29.2)	3 (13.6)	0.379
Alpha blocker	25 (19.0)	3 (8.1)	15 (20.8)	7 (31.8)	0.062
Diuretics	23 (17.5)	3 (8.1)^a^	12 (16.7)^ab^	8 (36.4)^b^	**0.030**
Number of agents, median (min–max)	1.6 (1.0–4.0)	1 (1–3)	1.5 (1–4)	2 (1–3)	0.050
Laboratory data					
FPG (mg/dL), median (min–max)	96 (56–375)	90 (77–130)^a^	96.5 (71–196)^b^	104 (56–375)^b^	**< 0.001**
Creatinine (mg/dL), median (min–max)	0.8 (0.6–2)	0.71 (0.6–1.4)^a^	0.89 (0.6–1.9)^b^	1.04 (0.7–2)^b^	**< 0.001**
e‐GFR (ml/min/1.73 m^2^), median (min–max)	93 (32–138)	115 (65–138)^a^	85.5 (32–114)^b^	65 (36–100)^c^	**< 0.001**
Uric acid (mmol/L), median (min–max)	5.9 (4.8–7.2)	5.35 (3.3–10.9)	6.1 (2.9–12.9)	6.55 (2.9–10.3)	0.059
Hemoglobin (g/dL), mean ± SD	13.8 (8.0–17.3)	14 ± 1.64	13.67 ± 1.99	13.55 ± 1.57	0.583
Albumin (g/L), median (min–max)	4.4 (2.0–5.4)	4.5 (3.7–5.4)	4.4 (2–5.1)	4.35 (3.9–4.6)	0.056
Sodium (mmol/L), median (min–max)	140 (132–150)	139 (133–145)	140 (134–147)	140 (132–150)	0.357
Potassium (meq/L), mean ± SD	4.4 (3.5–5.4)	4.38 ± 0.33	4.43 ± 0.4	4.61 ± 0.38	0.085
Calcium (mg/dL), median (min–max)	9.4 (6.6–10.7)	9.4 (8.5–10.5)	9.4 (8–10.7)	9.55 (6.6–10.6)	0.824
Phosphorus (mg/dL), median (min–max)	3.6 (2.5–7.1)	3.7 (2.5–6.2)	3.55 (2.7–5.2)	3.7 (2.8–7.1)	0.309
Intact PTH (pg/mL), median (min–max)	71.4 (5.6–192.4)	65 (25–190.4)	67.6 (24.6–171.1)	76.7 (5.6–192.4)	0.571
Ferritin (ng/mL), median (min–max)	66.7 (4.0–312.7)	65.94 (8.9–194.2)	60.2 (4–312.7)	87.7 (8.2–272.3)	0.939
Total cholesterol (mmol/L), mean ± SD	213 (56.6–306)	196.98 ± 54.47	223.08 ± 42.45	201.42 ± 53.07	0.110
LDL (mmol/L), mean ± SD	138 (60–215)	126.77 ± 32.92	141.21 ± 36.9	120.07 ± 42	0.157
Triglyceride (mmol/L), median (min–max)	153 (44–598)	138.5 (67–396)	156.5 (44–598)	149.5 (52–409)	0.565
Hs‐CRP (mg/dL), median (min–max)	0.3 (0.1–5.7)	0.3 (0.1–2.6)	0.3 (0–3.5)	0.73 (0.1–5.7)	0.089
Proteinuria (mg/gram), median (min–max)	114 (11–7209)	58.5 (11–2149)^a^	116.5 (23–7209)^b^	200 (54–1827)^b^	**0.006**

*Note:* Kruskal–Wallis test, chi‐squared test a–c: those groups that have the same or common letters do not differ significantly. IQR, interquartile range; ACEİ, ACE inhibitor; PTH, parathormone. Values in bold indicate statistical significance (*p* < 0.05).

Abbreviations: ARB, angiotensin receptor blocker; BMI, body mass index; CCB, calcium channel blocker; DM, diabetes mellitus; e‐GFR, estimated glomerular filtration rate; FPG, fasting plasma glucose; Hs‐CRP, high‐sensitivity C‐reactive protein; LDL, low‐density lipoprotein.

### 3.2. Overall 24‐h ABPM Findings

Twenty‐four‐hour ABPM data are summarized in Table 2. Median (IQR 25%–75%) nocturnal SBP and DBP decreased values were 6.2% (1.5–11.2) and 9.1% (3.6–13.9), respectively. Overall, 45.8% of patients were identified as diastolic dippers and 29.7% were systolic dippers. Median (IQR 25%–75%) 24‐h total ePWV was 7.2 (5.5–8.5) m/s; 28.2% were in the low ePWV subgroup (< 6 m/s), 55.0% in the moderate (6–9 m/s), and 16.8% in the high (> 9 m/s).

**TABLE 2:** 24‐h ambulatory blood pressure monitoring (ABPM) data: overall and across the PWV subgroups.

Median (IQR 25%–75%)	Total (*n* = 131)	Pulse wave velocity groups			*p* value
		Low (*n* = 37)	Medium (*n* = 72)	High (*n* = 22)	
24‐h BP (mm Hg)					
Systolic BP	127 (118–139)	120 (112.5–124.5)	130 (119.2–139)	140 (127.7–145.2)	**< 0.001**
Diastolic BP	80 (72–89)	72 (65.5–77.5)	84 (74.5–92)	82 (75.7–91.2)	**< 0.001**
Mean arterial pressure	102 (93–112)	93 (87.5–99)	106 (95.2–114)	108.5 (100.7–116.2)	**< 0.001**
Pulse pressure	47 (42–54)	46 (42–50)	45.5 (41.53.7)	52.5 (49.7–58.2)	**0.001**
Daytime BP (mm Hg)					
Systolic BP	128 (121–140)	121 (114–127)	131 (121.2–140)	140 (127.7–145.5)	**< 0.001**
Diastolic BP	81 (81–91)	74 (67–81.5)	86 (77.2–94)	86.5 (77.7–92.5)	**< 0.001**
Mean arterial pressure	103 (95–113)	94 (89.5–101.5)	106 (99–115)	109.5 (101.7–116.5)	**< 0.001**
Pulse pressure	47 (42–54)	45 (41–49.5)	45.5 (41–54.7)	51.5 (47.7–59)	**0.004**
Night‐time BP (mm Hg)					
Systolic BP	120 (110–137)	112 (105.5–118.5)	123.5 (113–136)	138.5 (129.2–146)	**< 0.001**
Diastolic BP	74 (65–84)	65 (57.5–74)	79 (68–87.7)	78 (72.5–94.2)	**< 0.001**
Mean arterial pressure	95 (85–108)	85 (79.5–92.5)	99.5 (88–108)	107 (97.5–114.5)	**< 0.001**
Pulse pressure	47 (41–55)	45 (40–52.5)	46 (41–50)	55.5 (50.7–63.2)	**< 0.001**
Nocturnal BP dipping status					
Systolic dipping (%)	6.2 (1.5–11.2)	8.6 (4.4–11.8)	5.9 (1.6–10.2)	2.2 (−4.7–6.4)	**0.004**
Diastolic dipping (%)	9.1 (3.6–13.9)	11.4 (6–18.6)	8.1 (3.3–12.7)	7.4 (−0.5–13)	**0.020**
Systolic dippers, *n* (%)	39 (29.7)	17 (45.9)	18 (25.0)	4 (18.1)	**0.033**
Diastolic dippers, *n* (%)	60 (45.8)	21 (56.7)	29 (40.2)	10 (45.4)	0.263
PWV (m/s)					
24‐h	7.2 (5.5–8.5)	5.1 (4.9–5.4)	7.3 (6.8–8.3)	10.2 (9.7–11.4)	**< 0.001**
Daytime	7.1 (5.6–8.6)	5.2 (4.8–5.4)	7.3 (6.8–8.3)	10.1 (9.8–11.3)	**< 0.001**
Night‐time	7.0 (5.5–8.4)	4.9 (4.8–5.1)	7.2 (6.5–8.1)	10.3 (9.5–11.9)	**< 0.001**

*Note:* Kruskal–Wallis test, chi‐squared test. IQR: interquartile range. Values in bold indicate statistical significance (*p* < 0.05).

Abbreviations: BP, blood pressure; PWV, pulse wave velocity.

### 3.3. 24‐h ABPM Data Across PWV Subgroups

Compared with the other ePWV groups, the high ePWV subgroup had significantly higher 24‐h, daytime, and night‐time SBP (an expected association given the SBP‐dependent calculation method), MAP, and pulse pressure; the low ePWV subgroup had significantly lower 24‐h, daytime, and night‐time DBP (*p* range 0.004 to < 0.001; Table 2).

High ePWV was associated with the lowest nocturnal SBP decline (median 2.2% vs. 5.9% in moderate ePWV and 8.6% in low ePWV, *p* = 0.004) and a lower rate of systolic dipping (18.1% vs. 45.9% and 25.0%, respectively, *p* = 0.033) (Table 2).

Low ePWV showed the greatest nocturnal DBP decline (median 11.4% vs. 8.1% in moderate ePWV and 7.4% in high ePWV, *p* = 0.020) and a nonsignificant trend toward higher diastolic dipper status (56.7% vs. 40.1% and 45.4%, respectively, *p* = 0.263) (Table 2).

### 3.4. Baseline Characteristics Across PWV Subgroups

Patients with high ePWV were older than those with low and moderate ePWV (median (min–max) 73 (60–86) vs. 24 (17–43) and 52 (37–65) years, *p* < 0.001) (a difference consistent with the age‐dependent calculation method) and used more antihypertensive medications (median (min–max) 2 (1–3) vs. 1 (1–3) and 1.5 (1–4), *p* = 0.05). Diuretic use was more common in the high ePWV group than in the low ePWV subgroup (36.4 vs. 8.1%, *p* = 0.030) (Table 1). The low ePWV subgroup had lower BMI than the moderate subgroup (median (min–max) 27 (20–48) vs. 31 (22–45.1) kg/m^2^), *p* = 0.005) and a lower diabetes rate than both moderate and high subgroups (2.7 vs. 27.7% and 31.8%, respectively, *p* = 0.001) (Table 1).

The high ePWV subgroup had higher median (min–max) FPG (104 (56–375) vs. 90 (77–130) and 96.5 (71–196) mg/dL, *p* < 0.001), creatinine (1.04 (0.7–2) vs. 0.89 (0.6–1.9) and 0.71 (0.6–1.4 mg/dL, *p* < 0.001), and proteinuria (200 (54–1827) vs. 58.5 (11–2149) and 116.5 (23–7209) mg/g, *p* = 0.006), but lower eGFR (65 (36–100) vs. 115 (65–138) and 85.5 (32–114) mL/min/1.73 m^2^, *p* < 0.001) than the low and moderate groups. No subgroup differences were seen for gender, smoking, or other laboratory features (Table 1).

### 3.5. Correlations Between 24‐h PWV Values and Continuous Variables

In simple correlations, 24‐h ePWV correlated positively with age (*r* = 0.958, *p* < 0.001) (intrinsic to the calculation method), BMI (*r* = 0.245, *p* = 0.005), FPG (*r* = 0.329, *p* < 0.001), creatinine (*r* = 0.409, *p* < 0.001), uric acid (*r* = 0.231, *p* = 0.017), and proteinuria (*r* = 0.332, *p* = 0.001), and negatively albumin (*r* = −0.242, *p* = 0.011) and eGFR (*r* = −0.650, *p* < 0.001) (Table 3).

**TABLE 3:** Correlates of 24‐h pulse wave velocity.

Predictor	Simple correlation^a^		Partial correlation^b^	
	*r*	*p*	*r*	*p*
Age (years)	0.958	< 0.001	—	—
BMI (kg/m^2^)	0.245	0.005	0.063	0.483
FPG (mg/dL)	0.329	< 0.001	−0.216	**0.014**
Creatinine (mg/dL)	0.409	< 0.001	−0.150	0.092
eGFR (mL/min/1.73 m^2^)	−0.650	< 0.001	0.127	0.153
Uric acid (mmol/L)	0.231	0.017	0.106	0.285
Albumin (g/L)	−0.242	0.011	−0.085	0.384
Hemoglobin (g/dL)	—	—	0.042	0.641
Intact PTH (pg/mL)	—	—	0.083	0.560
Ferritin (ng/mL)	—	—	−0.097	0.500
Total cholesterol (mmol/L)	—	—	0.243	**0.047**
LDL (mmol/L)	—	—	0.359	**0.004**
Triglyceride (mmol/L)	—	—	−0.011	0.928
hs‐CRP (mg/dL)	—	—	−0.220	**0.031**
Proteinuria (mg/g)	0.332	0.001	−0.066	0.527
DBP 24 h (mmHg)	0.412	< 0.001	0.001	0.990
DBP day (mmHg)	0.383	< 0.001	−0.047	0.595
DBP night (mmHg)	0.469	< 0.001	0.080	0.368
MAP 24 h (mmHg)	0.526	< 0.001	−0.015	0.868
MAP day (mmHg)	0.477	< 0.001	−0.061	0.495
MAP night (mmHg)	0.557	< 0.001	0.093	0.296
PP 24 h (mmHg)	0.39	< 0.001	−0.039	0.660
PP day (mmHg)	0.36	< 0.001	−0.059	0.508
PP night (mmHg)	0.392	< 0.001	0.041	0.648
Systolic dipping (%)	−0.295	0.001	−0.124	0.164
Diastolic dipping (%)	−0.255	0.003	−0.144	0.105

*Note:* Values in bold indicate statistical significance (*p* < 0.05).

Abbreviations: BMI, body mass index; eGFR, estimated glomerular filtration rate; FPG, fasting plasma glucose; hs‐CRP: high‐sensitivity C‐reactive protein; MAP, mean arterial pressure; PP, pulse pressure; PWV, pulse‐wave velocity; SBP/DBP, systolic/diastolic blood pressure.

^a^Simple correlation: Pearson correlations with raw estimated PWV (ePWV).

^b^Partial correlation: Correlations calculated between ePWV and other variables after strictly controlling for age, age^2^, and SBP.

ePWV was also positively correlated with 24‐h, daytime, and night‐time values of DBP, MAP, and pulse pressure with the strongest associations for MAP‐night (*r* = 0.557, *p* < 0.001) and MAP‐24 h (*r* = 0.526, *p* < 0.001), and inversely with systolic (*r* = −0.295, *p* = 0.001) and diastolic dipping (*r* = −0.255, *p* = 0.003) (Table 3).

However, after adjusting for age, age^2^, and 24‐h SBP, the partial correlation analysis revealed that ePWV was negatively correlated only with FPG (*r* = −0.216, *p* = 0.014) and hsCRP (*r* = −0.220, *p* = 0.031) and was positively correlated only with total cholesterol (*r* = 0.243, *p* = 0.043) and LDL (*r* = 0.359, *p* = 0.004) (Table 3).

## 4. Discussion

The study findings revealed the presence of arterial stiffness, as indicated by high ePWV (> 9 m/s) at oscillometric PWA, in 16.8% of patients with treated hypertension. Arterial stiffness was associated with increased age, more frequent use of combination therapy (especially diuretic use), lower eGFR values, and higher levels of FPG, creatinine, and proteinuria. 24‐h daytime and night‐time values for SBP, MAP, and pulse pressure were significantly higher in patients with arterial stiffness. The smallest decline in nocturnal SBP and a lower rate of systolic dipper status were also determined in these patients.

PWV in this study was estimated using the Mobil‐O‐Graph device, which derives values through an algorithm incorporating age and SBP. Accordingly, the strong correlation observed between ePWV and age is largely expected and reflects this computational dependence. While unadjusted analyses suggested associations between ePWV and clinical factors such as reduced systolic dipping, these findings must be interpreted with caution due to the inherent structure of the estimation model. To rigorously address this dependency, we performed a partial correlation analysis strictly controlling for age, age^2^, and SBP. In this adjusted analysis, apart from hs‐CRP, FPG, total cholesterol, and LDL, no clinical, metabolic, or ABPM parameters remained significantly associated with ePWV. This indicates that the apparent correlations observed in the unadjusted analysis were primarily driven by the algorithmic inputs (age and blood pressure) rather than independent vascular alterations.

A previous study assessing cfPWV in hypertensive subjects (treatment‐naïve or receiving stable antihypertensive treatment) observed significant relationships between various 24‐h ABPM parameters (24‐h and daytime SBP, and 24‐h, daytime, and night‐time pulse pressure), age and arterial stiffness [9]. However, while arterial stiffness was reported to rise progressively in line with blood pressure increases in that study, pulse pressure was identified as the leading determinant of arterial stiffness, and no significant correlation was determined between nocturnal dipping and arterial stiffness [9]. Other studies have described the prognostic significance of SBP and pulse pressure for aortic PWV, with no significant association between DBP and PWV in essential hypertension [27, 28].

In our cohort, ePWV correlated positively with DBP, MAP, and pulse pressure across 24‐h/daytime/night‐time indices and inversely with nocturnal dipping. However, after adjusting for age, age^2^, and 24‐h SBP, no significant relationships with 24‐h ABPM parameters persisted. Nevertheless, the low ePWV group (< 6 m/s) exhibited a distinct diastolic profile compared to the higher ePWV groups, characterized by significantly lower 24‐h, daytime, and night‐time DBP values and the greatest decline in nocturnal DBP, although the higher rate of diastolic dipper status in this group did not reach statistical significance.

In a study using oscillometric PWA via 24‐h ABPM, 301 participants were divided into three groups (healthy, controlled hypertension, and uncontrolled hypertension). The highest PWV values were observed in the uncontrolled hypertension group, with older individuals, those with prediabetes, and nondippers in each group also exhibiting higher PWV [29]. Age, blood pressure, and nocturnal indices were identified as independent predictors of PWV in the multivariate regression model [29].

Research has suggested that PWV is a global indicator of macro‐ and microcirculatory health, which in turn serves as a strong predictor of cardiovascular events in hypertensive patients, independently of traditional risk factors (SBP, FPG, total cholesterol, and BMI), as well as chronic kidney disease progression, and the incidence of glucose intolerance or diabetes [4, 5, 30–32]. Notably, in our cohort, significant associations of ePWV with FPG, total cholesterol, LDL, and hs‐CRP were noted after correlation analysis adjusted for age, age^2^, and SBP.

Evidence suggests that nondipper status has no effect on PWV values in patients with controlled hypertension due to the cardiovascular protective effects of antihypertensive medications and good blood pressure regulation [29, 33, 34]. The low rate of systolic dippers (∼30%) in our cohort may appear high relative to general population estimates; however, similar or higher nondipping rates have been reported in treated hypertensive patients [35], those with chronic kidney disease (32%–68%) [36], certain ethnic groups (up to 68%) [37], and in large ABPM registries (> 40%) [38]. Thus, the observed prevalence is consistent with existing data for comparable clinical populations. While our findings suggest a preponderance of a nondipper pattern among individuals with higher ePWV values, it is important to note that dipping status is often correlated with SBP levels, which are themselves components of the ePWV estimation algorithm. Therefore, given that this association did not persist after controlling for algorithmic inputs, it appears to be driven by the device’s inherent computational dependence on blood pressure. This potential overlap should be considered when interpreting nocturnal dipping as an independent predictor of arterial stiffness in treated hypertensive patients [29, 39–41].

Significant negative correlation was observed between ePWV and eGFR in the present study, in addition to the positive correlations between ePWV and serum creatinine and proteinuria. This aligns with previous research linking kidney damage and impaired renal function via structural and functional alterations in the arterial wall to arterial stiffness [42–44]. However, these correlations did not persist after adjusting for age, age^2^, and systolic BP.

The significant correlation between ePWV and BMI in the present study supports the well‐established associations reported between BMI and arterial stiffness, which has been linked to increased mechanical strain on the arterial wall and the presence of obesity‐related metabolic abnormalities [45, 46]. However, this relationship lost statistical significance when the algorithmic effects were controlled. Regarding subgroup analysis, the low ePWV subgroup had lower BMI than the moderate subgroup.

The higher FPG values and higher rate of comorbid diabetes in our high ePWV subgroup with treated hypertension are particularly noteworthy since elevated PWV values have previously been described as predictors of new‐onset type 2 diabetes in hypertensive patients, independently of the effects of antihypertensive therapy [29, 47]. Hypertensive diabetic patients were reported to have significantly higher central blood pressure, peripheral blood pressure, and PWV than hypertensive nondiabetic patients in a previous study [8]. Our study also found a relationship between FPG and ePWV, independent of algorithmic effects.

Previous studies have suggested that the effects of drugs (e.g., peak and trough effects) have a greater impact on the variance in office readings compared to that in 24‐h ambulatory readings, while 24‐h ABPM also predicts the risk of cardiovascular events even after adjustment for classic risk factors including office blood pressure [48, 49]. Further studies are therefore needed to investigate whether the cost–benefit ratio favors adding ABPM with oscillometric PWV to the standard care of patients with treated hypertension, in the light of the clinical relevance of such an approach in terms of better monitoring of the progression of hypertension and improved cardiovascular risk assessment [48–50].

We studied a real‐world‐treated hypertensive cohort using standardized 24‐h ABPM with rigorous QC and comprehensive biochemical/renal phenotyping, and we report both raw and age, age^2^, SBP‐adjusted associations, enhancing interpretability and reproducibility; however, limitations include the cross‐sectional, single‐center design (limiting causal inference and generalizability), a modest sample size (*n* = 131) with reduced power for weaker effects, potential confounding from type 2 diabetes and active smoking (though smoking did not differ across ePWV subgroups and diabetes was not an independent determinant), self‐reported medication adherence, the algorithmic dependence of ePWV on age, age^2^ and SBP (which may inflate correlations and limit independence), and lack of individual sleep‐pattern data. Addressing these constraints in future studies will refine understanding of PWV determinants and their role in hypertension management.

## 5. Conclusions

In patients with treated hypertension, elevated ePWV co‐occurs with markers of vascular and metabolic burden, including older age, kidney impairment, higher blood pressure, and nondipping BP patterns. However, after accounting for the embedded mathematical effects of age, age^2^, and SBP, the statistical significance of these correlations did not persist. These findings suggest that oscillometric ePWV functions primarily as a composite marker reflecting its algorithmic inputs, rather than providing a distinct biological signal independent of age and blood pressure. Therefore, while oscillometric ABPM remains a valuable tool for blood pressure monitoring, ePWV values should be interpreted with awareness of their strong mathematical dependence, as they may not offer additional diagnostic information beyond these established risk factors in a cross‐sectional setting.

## Author Contributions

Damla Tufekci and Ekrem Kara contributed to the conception/design of the research; Ekrem Kara contributed to the acquisition, analysis, and interpretation of the data; Damla Tufekci drafted the manuscript; Tuncay Sahutoglu critically revised the manuscript; and Damla Tufekci agreed to be fully accountable for ensuring the integrity and accuracy of the work.

## Funding

The authors declare that no funds, grants, or other support were received during the preparation of this manuscript.

## Disclosure

All authors read and approved the final manuscript.

## Ethics Statement

This study was approved by the Recep Tayyip Erdogan University‐Clinical Research Ethics Committee (Date of Approval: 11/07/2018; Protocol no: 148).

## Consent

Written informed consent was obtained from all participants.

## Conflicts of Interest

The authors declare no conflicts of interest.

## Data Availability

The data that support the findings of this study are available on request from the corresponding author.
